# Relationship between salivary neuropeptide Y and performance under stress: a study on police firearms officers

**DOI:** 10.3389/fnhum.2026.1753205

**Published:** 2026-05-01

**Authors:** Marc V. Jones, Chris Murgatroyd, Nathan Smith, Lucy Walker, Martin James Turner, Andrew McCann, Elizabeth C. Braithwaite, Danielle Burns, Maxted Neal, Paul Emmerson, Leonie Webster, Martin Jones

**Affiliations:** 1 School of Psychology, Manchester Metropolitan University, Manchester, United Kingdom; 2 Department of Life Sciences, Manchester Metropolitan University, Manchester, United Kingdom; 3 Centre for Trust, Peace and Social Relations, Coventry University, Coventry, United Kingdom; 4 Hult International Business School, London, United Kingdom; 5 Cervus Defence and Security Ltd., Bristol, United Kingdom; 6 Human Sciences Group, CBR Division, Defence Science and Technology Laboratory, Salisbury, United Kingdom

**Keywords:** anxiety, pressure, resilience, selection, shooting performance, NPY, salivary biomarkers, law enforcement

## Abstract

**Introduction:**

An emerging body of evidence suggests that plasma neuropeptide Y (NPY) concentration is positively associated with performance under conditions of psychological stress. However, whether NPY concentrations in saliva are associated with performance under stress remains unknown.

**Methods:**

We collected saliva samples in a high-performance environment and tested the associations between salivary NPY, self-reported psychological variables, and performance during a stressful task. Participants were 15 male police firearms officers (M_age_ = 33.57 years, *SD* = 3.92) enrolled in a demanding selection course. Participants provided their saliva samples using the passive drool method 1 h before the shooting task, which they needed to pass to remain on the course (high-stress condition). Additionally, they provided a sample on a low-stress training day (low-stress condition). Self-reported measures of psychological state included measures of state anxiety (cognitive and somatic), self-confidence, and threat appraisals provided 1 h prior to the shooting task.

**Results and discussion:**

NPY concentrations were detected under both high- and low-stress conditions. Mean NPY levels (ng/mL) were higher before the high-stress shooting task (*M* = 1.56, *SD* = 0.88) than on the low-stress training day (*M* = 1.13, *SD* = 0.52), but this difference did not reach significance. Salivary NPY concentrations prior to the shooting task were positively and strongly associated with shooting performance (*n* = 13, *r* = 0.71, *p* = 0.007). There were no associations between psychological variables and salivary NPY or shooting performance. This study provides an important initial step in exploring the salivary NPY–performance relationship. Further studies are needed to determine whether these results can be replicated across different performance tasks and domains.

## 1 Introduction

Defence and security personnel are often required to complete physically, psychologically, and socially demanding tasks. Resilient performance when completing these tasks is described as the maintained or improved execution of competence and is associated with a range of biopsychosocial factors (Jones et al., 2022a). One biomarker that shows promise in understanding resilient performance is neuropeptide Y (NPY). NPY is a 36-amino-acid peptide that is widely distributed in both the central and peripheral nervous systems. In the brain, NPY expression is particularly high in several regions, including the hypothalamus, septum, nucleus accumbens, periaqueductal grey, locus coeruleus, amygdala, hippocampus, cerebral cortex, basal ganglia, and thalamus (Kask et al., 2002). Functioning as a neurotransmitter, NPY impacts several biological processes, including stimulating food intake, regulating circadian rhythms, and, importantly, dampening stress-related anxiety effects (Reichmann and Holzer, 2016).

A consistent body of evidence demonstrates that endogenous NPY produces stress-reducing effects in animals (Hirsch and Zukowska, 2012; Reichmann and Holzer, 2016). Emerging studies suggest similar effects in humans. A recent systematic review and meta-analysis reported that NPY levels were significantly lower in the plasma and cerebrospinal fluid of patients with post-traumatic stress disorder (Tural and Iosifescu, 2020). Patients with major depressive disorder also had significantly lower levels of NPY in the plasma (but not in the cerebrospinal fluid) compared to controls. These findings suggest that circulating NPY may be a marker of the psychological functioning of an individual.

In addition to its observed relationship with mental health, it is reasonable to hypothesize that NPY may play an active role in resilient performance in stressful environments (Meijen et al., 2020). Centrally, NPY is involved in triggering the hypothalamic–pituitary–adrenal (HPA) axis via activation of the hypothalamus (Krysiak et al., 1999) and later proposed to act as a brake to inhibit excessive activation of the stress response (Hirsch and Zukowska, 2012). The later release of NPY occurs through a distributed network of circuits utilizing NPY signalling, including from the amygdala and hippocampus, and takes place after the release of corticotropin-releasing hormone (CRH), with NPY terminating the stress response by counteracting the actions of CRH (Heilig, 2004). In the periphery, under sympathetic activation, NPY is co-released with norepinephrine (NE) from axon terminals and enhances the vasoconstriction effect of NE but also inhibits the further release of NE. Overall, NPY may enable a necessary short-term stress response but prevent costly long-term activation of stress (Heilig, 2004). Thus, in performance environments, higher NPY may be associated with a more adaptive stress response because it inhibits the duration of time for which cortisol may be elevated and potentially has detrimental effects on components of successful performance, such as working memory (Shields et al., 2015) and decision-making (Wemm and Wulfert, 2017).

Plasma NPY increases under psychological stress in military populations (Morgan et al., 2002; Morgan et al., 2000) and is positively associated with performance under stress (Morgan et al., 2000). However, previous studies relied on the collection and analysis of blood samples to determine NPY levels, which can be more difficult to achieve in performance environments (e.g., requiring trained individuals to collect blood samples). An alternative approach is to quantify NPY concentrations in saliva samples collected *in situ*. NPY is released from nerve endings in the submandibular, sublingual and parotid glands in the mouth and is involved in the regulation of salivary secretion (Ekström et al., 1996). Although it is possible to detect NPY in saliva, there has been limited research on this topic in humans (Kluess et al., 2019; Şemsi et al., 2023), and to our knowledge, no study has explored its association with skilled performance. Furthermore, a previous study involving a sample of 14 women suggested that NPY levels in the plasma and saliva were not correlated (Kluess et al., 2019). This may be due to differences in the release sites of NPY in the plasma (e.g., peripheral sympathetic nerve fibres and adrenal medulla) compared to saliva (e.g., produced locally in the submandibular, sublingual, and parotid glands in the mouth). Therefore, while there may not be an association between NPY in the plasma and saliva, an increase in salivary NPY is observed under stress (Şemsi et al., 2023), and salivary NPY may still be a valid measure of stress resilience.

There are novel implications if salivary NPY is validated as a biomarker of psychological stress resilience in performance settings. For example, it could be used as a more objective (compared to self-report) way to monitor and assess performance readiness during critical time periods (e.g., before a high-risk operation). To determine whether salivary NPY can be used as a biomarker of stress resilience in high-stress performance settings, saliva samples were collected for NPY analysis, and self-reports were obtained from police firearms officers prior to completing a stressful shooting task, which participants needed to pass to remain on a selection course. Saliva samples for NPY were also collected on a low-stress training day (low-stress condition). Our hypotheses were:

1. Higher levels of salivary NPY prior to the shooting task are associated with higher performance scores on the shooting task.
2. Salivary NPY is higher before the shooting task (high-stress condition) than on a low-stress training day (low-stress condition).
3. An increase in salivary NPY levels between the shooting task (high-stress condition) and low-stress training day (low-stress condition) is positively associated with performance on the shooting task.
4. Salivary NPY collected before the shooting task is positively associated with pre-shooting task self-reported psychological measures of self-confidence and negatively associated with cognitive anxiety, somatic anxiety, and threat appraisals.

## 2 Methods

### 2.1 Design

This was a quantitative, observational, repeated-measures study, with data collected *in situ* at a defence and security training facility.

### 2.2 Participants

For this study, all police officers who enrolled in a firearms course to join an advanced firearms officer cadre were identified as potential participants. This participant group was part of a larger project exploring resilient performance in armed police and Royal Air Force personnel (Jones et al., 2022b). All police officers enrolled in the course received an email prior to the course, alerting them to the study and orienting them to the research, along with a copy of the participant information sheet (PIS), outlining the study, and an informed consent form. When the police officer arrived to commence the course, they received detailed information about the study in a presentation given by MVJ and AM, paper copies of the PIS, and an informed consent form. The time between first receiving the PIS form (via email) and being invited to provide consent to participate was a minimum of 24 h. Any queries or concerns from the participants were addressed by the researchers during the presentation, and they gave their consent to participate individually.

A total of 15 police officers participated in the study and represented the entire cohort of participants who underwent a selection course in this hard-to-reach population. Although modest in size, the sample comprised individuals accustomed to performing under stress who were currently working as armed police and had achieved high-performance standards to be selected for the course. Participants were therefore both skilled and experienced and were engaged in a personally meaningful task, one in which performance was important for career progression to an advanced firearms officer cadre. Thus, this is a unique, rarely studied, skilled, and experienced population of the police force to explore the effects of stress on performance.

### 2.3 Protocol

Ethical approval was granted for the present study by the Ministry of Defence (2072/MODREC/21) and Manchester Metropolitan University (37660) Research Ethics Committees. Data were collected from a shooting-based performance task that occurred on the first day of an assessment and selection course (high-stress condition), and 28 days later, participants provided a sample on a low-stress training day (low-stress condition). For the high-stress data collection, 1 h prior to the shooting task, saliva samples were collected between approximately 11.30 a.m. and 11.55 a.m., and self-report measures were completed. During low-stress data collection, saliva samples were taken at approximately 10.00 a.m. While there were slight differences in the time saliva samples were taken, this was done to fit the demands of the course. This marginal difference in timing was not expected to affect the findings. Currently, there is limited knowledge regarding the diurnal variation of NPY in saliva, and previous research has found no significant NPY variation (Baker et al., 2013). Other studies have suggested that plasma NPY has two peaks, one earlier at ~8 a.m. and one later at ~4 p.m. (Löckinger et al., 2004), with troughs between ~10 a.m. and 12 p.m. and ~8 p.m. and ~10 p.m.

### 2.4 Measures

#### 2.4.1 Salivary NPY

Saliva samples were obtained using the passive drool method. Participants were asked to refrain from consuming food and drinks for 1 h prior in order to provide saliva samples. The samples were immediately frozen at −18 °C for transportation to Manchester Metropolitan University. On arrival, the samples were defrosted, and ~1 mL of saliva from each sample was centrifuged at 2,000 rpm for 5 min, and the acellular supernatant was aliquoted into separate 100 μL samples that were stored at −80 °C until analysis. Aliquots were not subjected to freeze–thaw cycles. Concentrations of NPY were determined using an enzyme-linked immunosorbent assay (ELISA). Sample processing was accomplished using commercially available neuropeptide (Phoenix Pharmaceuticals, Inc., CA, USA) kits. Measurements were performed in duplicate following the kit instructions, diluting samples 1:1 with assay buffer (Kluess et al., 2019). The optical density of the samples and NPY standards was measured by a microplate reader (The FLUOstar Omega from BMG Labtech, Germany) at 450 nm, according to the relevant standard curves, and the mean of the two replicates was calculated for each sample.

#### 2.4.2 Psychological measures

Self-reported psychological measures were used to collect information about stress responses. These were part of a number of measures collected in a larger project exploring resilient performance in armed police and Royal Air Force personnel (Jones et al., 2022b).

##### 2.4.2.1 Anxiety and self-confidence

The Mental Readiness Form (MRF)-Likert (Krane, 1994) has three 11-point scales in which participants indicated how they felt in response to the upcoming shooting task. The item prompts were as follows: my thoughts are (assessing cognitive anxiety) with 1 = not worried, 11 = worried; my body feels (assessing somatic anxiety) with 1 = not tense, 11 = tense; and I am feeling (assessing confidence) with 1 = confident, 11 = scared.

##### 2.4.2.2 Demands and resources

Demand and resource evaluations were measured using the 11-item challenge and threat scale (Mendes et al., 2007). Participants indicated how they were feeling in relation to the upcoming shooting task on a series of items, measuring both demands (six items) and resources (five items) on a 6-point scale ranging from 1 (strongly disagree) to 6 (strongly agree). The challenge and threat index was calculated by subtracting the average score for demands from the average score for resources. Positive numbers indicate a challenge state, and negative numbers indicate a threat state.

#### 2.4.3 Shooting performance

Participants were already qualified firearms officers and completing the assessment and selection course to achieve promotion to an advanced cadre. To be eligible for selection to this advanced cadre, they had to be skilled and competent in their current role as armed officers. They completed a shooting-based task at a specialist indoor range, which included a series of target-based live-fire shooting rounds with and without ventilator masks. Participants used two different weapons (pistol and rifle) at ranges between 5 m and 25 m with a mixture of stationary and mobile targets. Participants were engaged in the shooting task in groups (typically with five participants working simultaneously); however, the task (e.g., targets to aim for) was individual. Some shooting tasks involved performing under physical fatigue, whereas others involved changing weapons. Participants’ performances were scored on a scale of 0 to 100%, based on shooting accuracy. The task was completed on the first day of the course and was used to determine progression to the remainder of the advanced training course. Therefore, the performance outcome was inherently important to each individual, and this shooting task was considered one of the more stressful parts of the course.

### 2.5 Data analysis

Data were initially assessed for normality. For the high-stress qualification shoot of the 15 participants, 14 participants completed the psychological measures, and 13 participants provided usable saliva samples. Two samples could not be processed for NPY analysis owing to the quality of the saliva sample produced. On the low-stress training day, all participants (*N* = 15) provided usable saliva samples. A difference score for NPY (NPY difference) was calculated (NPY high-stress qualification shoot—NPY low-stress training day). Psychological and performance data were normally distributed, as assessed via Shapiro–Wilk tests, histograms, and quantile–quantile (Q–Q) plots. NPY data from the high-stress qualification shoot and low-stress training day were non-normally distributed with a negative skew; therefore, non-parametric tests were employed in the analyses using the NPY data. A Wilcoxon signed-rank test was used to compare NPY scores in the high-stress qualification shoot with those from the low-stress sample. One-tailed Spearman’s correlations were used to examine associations between salivary NPY in the high-stress qualification shoot, NPY in the low-stress training day, NPY difference between the two measures, and psychological variables and shooting performance, whereas two-tailed Pearson correlations were used to assess associations between self-reported psychological variables and shooting performance (Table 1). There was statistical power (>0.80) to detect large effects. Data were analysed using IBM SPSS Statistics, version 28.0.1.1 (15).

**Table 1:** Correlations between salivary NPY, psychological measures, and shooting performance.

Measure	M (SD)	Shooting performance	Cognitive anxiety	Somatic anxiety	Self-confidence	Challenge and threat index	NPY qualification shoot	NPY low-stress training day
Shooting performance	95.07 (2.06)							
Cognitive anxiety	7.43 (2.44)	−0.41						
Somatic anxiety	6.64 (2.37)	−0.42	0.43					
Self-confidence	5.64 (1.65)	−0.02	0.46	0.28				
Challenge and threat index	1.39 (0.70)	0.42	−0.75*	−0.37	−0.03			
NPY qualification shoot	1.56 (0.88)	0.71*	−0.02	−0.31	0.07	0.05		
NPY low-stress training day	1.16 (0.55)	0.15	0.39	−0.07	0.10	−0.13	0.34	
NPY difference	0.43 (0.78)	0.40	−0.07	0.04	−0.08	0.13	0.74**	−0.21

*p < 0.05, **p < 0.001. *n* = 14 for psychological variables and shooting performance; *n* = 13 for NPY. Mental readiness-confident: Higher scores indicate lower confidence.

## 3 Results

**Figure 1:**
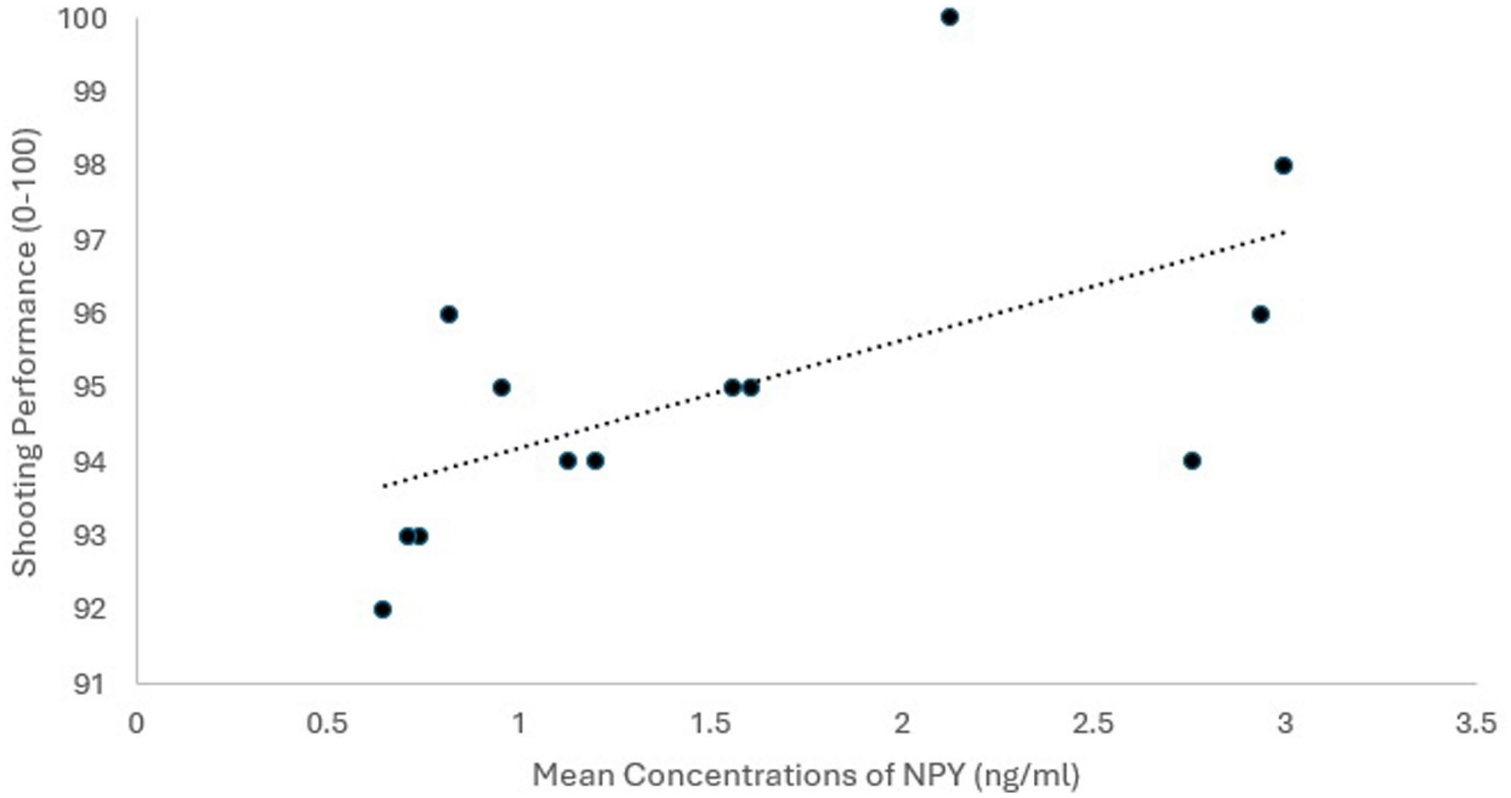
Association of salivary NPY with shooting performance scores. *N* = 13, shooting performance scores ranged from 92 to 100.

Participants (*N* = 15) were men with a mean age of 33.57 years (*SD* = 3.91, range = 25–41). We detected NPY in saliva samples collected *in situ* in the high-stress shooting task (*M* = 1.56 ng/mL, *SD* = 0.88, *n* = 13) and on a low-stress training day (*M* = 1.16 ng/mL, *SD* = 0.55, *n* = 15). The intra-assay coefficient of variation was 1.198%. All analyses involving NPY only included the 13 participants who provided usable samples on both occasions. NPY levels were higher in samples collected prior to the high-stress shooting task than in those collected during the low-stress condition, with a medium effect of Cohen’s *d* = 0.55 (Cohen, 1992) and a 95% confidence interval of [−0.05, 1.12], reflecting the small sample size. This difference was not significant at a conventional alpha level using a Wilcoxon signed-rank test, W = 25, *p* = 0.152, where the median NPY score was 1.2 in the high-stress condition and 0.89 in the low-stress condition. Salivary NPY collected prior to the qualification shoot was strongly associated with shooting performance (*r* = 0.71, *p* = 0.007) (see Figure 1). Participants performed well in the shooting task, with performance scores ranging from 92 to 100.

The difference in NPY concentration between the high-stress and low-stress conditions did not predict shooting performance (*r* = 0.40, *p* = 0.09). There were no significant associations between the psychological variables and NPY in the high-stress condition, NPY in the low-stress condition, NPY difference score, and shooting performance (Table 1). The challenge and threat index was positive (i.e., above 0), indicating that, on average, perceived resources were greater than perceived demands, reflecting the skilled and experienced nature of the participants. Each participant reported higher scores for resources than for demands. The only significant association between the psychological variables was negative between the challenge and threat index and cognitive anxiety. Participants with greater challenge appraisals reported lower levels of cognitive anxiety.

## 4 Discussion

This study provides initial evidence that saliva samples can be collected and used to quantify NPY concentrations in a skilled defence and security population, in this case, police firearms officers, *in situ,* and that these concentrations are associated with better performance on a subsequent stressful task. This is the first study to report NPY concentrations measured in saliva in a performance environment and to relate NPY levels to subsequent performance.

Collecting data from defence and security operators, especially in dynamic situations, such as on assessment and selection courses, is helpful to explore resilient performance under stress. Particularly when, as in this sample, participants were using live rounds for the shooting task, which added to the complexity of gathering research data. The data from this study suggest that sampling NPY from saliva samples can be a useful methodological approach and, for some groups, may be seen as less invasive and more economical than attempting to collect blood samples. Furthermore, given that there are differences in the release site for NPY in plasma (e.g., peripheral sympathetic nerve fibres, adrenal medulla) compared to saliva (e.g., produced locally in the submandibular, sublingual, and parotid glands in the mouth), NPY in saliva may provide a complementary perspective on NPY and resilience to that observed with NPY in plasma. The present findings show that salivary NPY collected prior to the high-stress qualification shoot was positively associated with shooting performance. In other words, participants with higher concentrations of salivary NPY performed better on the high-stress shooting task, and these findings represented a large effect. This finding aligns with previous studies in defence and security settings (Morgan et al., 2000), which identifies an association between NPY resilient performance. However, unlike Morgan et al., absolute NPY levels, rather than NPY difference scores (NPY high-stress qualification shoot—NPY low-stress training day), were related to performance. However, the association between NPY difference and performance was in the direction predicted and represented a medium effect size, which is in line with the previous findings of Morgan and colleagues. Furthermore, NPY levels were not significantly higher in the high-stress shooting task compared to the low-stress training day, although again the differences were in the direction hypothesized and represented a medium effect size. While there are some differences in our findings from those of previous studies (e.g., Morgan et al., 2000), our findings do complement this research and point to the possible role of NPY in stress resilience in performance contexts. This understudied area requires further investigation, and future research could explore whether larger samples show significant increases in salivary NPY that are associated with resilient performance. Alternatively, it may be that baseline levels of salivary NPY are more strongly associated with stress resilience, reflecting something that can be accessed when required under high stress and reflecting a trait resilience to psychological stress in line with previous findings on NPY in plasma and mental health (Tural and Iosifescu, 2020). It is worth noting that cross-study comparisons can be complicated by differences in the salivary concentrations of NPY. For example, the levels of salivary NPY are noticeably higher in our sample than in some previous studies (Kluess et al., 2019) and noticeably lower than others (Şemsi et al., 2023). There is variation across studies, which could be driven by individual differences, including biological sex, differences in sampling methods, and different ELISA kit specifications.

Salivary NPY was not significantly associated with any of the self-reported measures, which is in line with some previous studies that have not found associations between physical stress responses and psychological self-reports (Meijen et al., 2014). Although widely used in stress research, it is possible that self-reports may not reflect consciously available situational evaluations and, in particular, processes that may occur unconsciously, such as the immediate evaluation of a stressor (LeDoux, 1998). Saliva samples were collected 1 h before performance, and future research examining associations between salivary NPY, self-reported psychological state measured at multiple time points, and performance outcomes could identify optimal sampling time points that have practical applications in high-demand settings. However, there were some associations between the self-reported measures. Specifically, there was a significant negative association between the challenge and threat index and cognitive anxiety. There was also a medium effect size, albeit a non-significant, negative correlation between cognitive anxiety and performance, somatic anxiety and performance, and NPY and somatic anxiety.

This is the first study exploring the association between concentrations of salivary NPY measured and skilled performance; however, it has limitations. The most notable limitation is the sample size. Although the sample size was modest, the data were collected from the entire sample available in a hard-to-reach and hard-to-access population comprising individuals accustomed to performing under stress and who were currently working as armed police. Also, the participant sample were men (while it is important to recognize that female armed response officers have passed the course and serve in the cadre, this is not typical of these cohorts), and we may observe different results in female participants. The participants were also engaged in a personally meaningful task (one in which performance was important for career progression) and were skilled at the task. Therefore, they were considered to be an ideal group to study the effects of stress on performance. As a high-performing population, there would be homogeneity in performance levels; therefore, variations in performance could be more easily attributed to the stress of the situation. Although we had no baseline measure of performance, all participants had successfully performed comparable tasks in the past and had scored sufficiently high to qualify for the assessment and selection course. While there were ceiling effects and all participants performed with >92% shooting accuracy, a little more variability would have been beneficial. The sample was modest; however, it was similar in size to previous research on NPY (Kluess et al., 2019). There were also minor variations in the timings of the NPY assessment between the high-stress shooting test and the low-stress training day. While some research (Löckinger et al., 2004) has suggested that there is diurnal variation in NPY, we collected data during a trough between ~10 a.m. and 12 p.m. This timing indicates that NPY levels are lower and less variable during this period. In addition, the saliva samples were collected 1 h before the shooting task, as we were aware of the importance of the task for the participants and wanted to avoid interfering with their normal mental and physical preparation (e.g., checking their equipment). However, this could be a possible limitation, as participants were likely not experiencing the same level of stress at this time as they were closer to, or during, the shooting task itself. The findings would have been more robust if samples had been taken immediately prior to the commencement of the task. Other limitations include the lack of control biomarkers, such as cortisol or a measure of NPY in plasma. These biomarkers may have helped elucidate differences in the stress response across different performance outcomes. This could have pointed to potential pathways to explain why salivary NPY was associated with better performance under stress. Overall, while the modest sample size means that the findings need to be viewed cautiously and are not generalisable, the direction and strength of effects suggest a promising avenue for future research.

The present data are the first step in illustrating the association between salivary NPY and resilient performance. The ability to use an easy-to-collect saliva-based biomarker to explore associations with behaviour, specifically performance levels, under stress, could be a helpful tool in determining performance readiness. If this finding is replicated in future studies with larger samples, it could be informative for applied practice in monitoring and enhancing human performance under stress. Elucidating how salivary NPY relates to brain NPY levels and brain activity under stress is also important, as is discerning the relationships between salivary NPY and perceptual experiences. Additionally, future studies should aim to repeatedly measure salivary NPY to determine its dynamic nature in response to physical and psychological challenges. Recently, there have been novel developments in which the analysis of NPY in sweat allows real-time measures throughout the day (Churcher et al., 2022). These developments could be relevant for gathering repeated assessments of NPY, particularly in populations that are typically difficult to study, such as defence and security operators. Collecting and analysing NPY in (near) real-time would allow for more dynamic investigations of its role in performance and could be used to monitor and potentially predict resilient performance.

## Data Availability

The datasets presented in this article are not readily available due to security restrictions surrounding the sample, therefore the supporting data cannot be made available.
